# Genome-wide identification and transcriptome profiling reveal that E3 ubiquitin ligase genes relevant to ethylene, auxin and abscisic acid are differentially expressed in the fruits of melting flesh and stony hard peach varieties

**DOI:** 10.1186/s12864-019-6258-0

**Published:** 2019-11-21

**Authors:** Bin Tan, Xiaodong Lian, Jun Cheng, Wenfang Zeng, Xianbo Zheng, Wei Wang, Xia Ye, Jidong Li, Zhiqian Li, Langlang Zhang, Jiancan Feng

**Affiliations:** 1 College of Horticulture, Henan Agricultural University, Zhengzhou, 450002 China; 2 Henan Key Laboratory of Fruit and Cucurbit Biology, Zhengzhou, 450002 China; 3 Zhengzhou Fruit Research Institute, Chinese Academy of Agricultural Sciences, Zhengzhou, 450009 China

**Keywords:** Ubiquitin protein ligase gene family, The UPS pathway, Peach (*Prunus persica*), Flesh texture, Fruit ripening

## Abstract

**Background:**

Ubiquitin ligases (E3) are the enzymes in the ubiquitin/26S proteasome pathway responsible for targeting proteins to the degradation pathway and play major roles in multiple biological activities. However, the E3 family and their functions are yet to be identified in the fruit of peach.

**Results:**

In this study, genome-wide identification, classification and characterization of the E3 ligase genes within the genome of peach (*Prunus persica*) was carried out. In total, 765 E3 (PpE3) ligase genes were identified in the peach genome. The PpE3 ligase genes were divided into eight subfamilies according to the presence of known functional domains. The RBX subfamily was not detected in peach. The PpE3 ligase genes were not randomly distributed among the 8 chromosomes, with a greater concentration on the longer chromosomes. The primary mode of gene duplication of the PpE3 ligase genes was dispersed gene duplication (DSD). Four subgroups of the BTB subfamily never characterized before were newly identified in peach, namely BTBAND, BTBBL, BTBP and BTBAN. The expression patterns of the identified E3 ligase genes in two peach varieties that display different types of fruit softening (melting flesh, MF, and stony hard, SH) were analyzed at 4 different stages of ripening using Illumina technology. Among the 765 PpE3 ligase genes, 515 (67.3%) were expressed (FPKM > 1) in the fruit of either MF or SH during fruit ripening. In same-stage comparisons, 231 differentially expressed genes (DEGs) were identified between the two peach cultivars. The number of DEGs in each subfamily varied. Most DEGs were members of the BTB, F-box, U-box and RING subfamilies. PpE3 ligase genes predicted to be involved in ethylene, auxin, or ABA synthesis or signaling and DNA methylation were differentially regulated. Eight PpE3 ligase genes with possible roles in peach flesh texture and fruit ripening were discussed.

**Conclusions:**

The results of this study provide useful information for further understanding the functional roles of the ubiquitin ligase genes in peach. The findings also provide the first clues that E3 ligase genes may function in the regulation of peach ripening.

## Background

The ubiquitin/26S proteasome pathway in eukaryotes affects nearly all aspects of growth and development [1] through the regulated degradation of proteins. The ubiquitin/26S proteasome pathway consists of three key enzymes, namely an E1 (the ubiquitin-activating enzyme), an E2 (the ubiquitin-conjugating enzyme) and an E3 (the ubiquitin ligase). An E3 protein is responsible for selecting and then transferring the E2-bound ubiquitin to the target protein. The number of E1s, E2s and E3s varies greatly among various plants. For example, *Arabidopsis* has two E1s, 47 E2s and more than 1300 potential E3s. Rice has six E1s, 49 E2s and more than 1300 E3s [2]. E3 proteins are the most numerous components within the ubiquitin/26S proteasome pathway, due to their role in interacting with the multitude of targets in the plant.

The E3 superfamily comprises single subunit proteins and cullin-based multi-subunit protein complexes, both of which can mediate ubiquitination [3, 4]. Different E3s recognize specific substrates. The nine E3 subfamilies include the RING, HECT, U-box, F-box, cullin, BTB, DDB, RBX and SKP subfamilies. Members of the RING, HECT and U-box subfamilies can function as a single subunit, while SCF (SKP1, Cullin1 and F-box protein), CUL3-BTB and CUL4-DDB proteins function in multi-subunit protein complexes. In these complexes, the F-box or BTB proteins recognize substrates. Many of the subfamilies of the E3 proteins have been extensively studied, see the reviews [5–7].

The establishment of the plantsUPS (a database of plant proteins related to the ubiquitin proteasome system) enables the exploration and comparative analysis of E3 ligase genes in higher plants (http://bioinformatics.cau.edu.cn). The genome-wide identification and classification of E3 ligase genes is already available in a few plants, including Arabidopsis (*Arabidopsis thaliana*), rice (*Oryza sativa*), poplar (*Populus trichocarpa*), soybean (*Glycine max*), grape (*Vitis vinifera*), *Medicago truncatula*, and maize (*Zea mays*) [2].

Peach (*Prunus persica* L.) is a major commercial stone fruit grown worldwide, with its center of origin in China. Fruit texture is an important characteristic of peach. Fruit texture affects not only peach storage and processing, but also consumer preference. Peach is a climacteric fruit that undergoes changes in ethylene production and firmness during ripening. According to the changes in fruit firmness and texture during ripening, peach cultivars are divided into three groups, including melting flesh (MF), non-melting flesh (NMF) and stony hard (SH) [8]. Previous studies have shown that endopolygalacturonase (endo-PG), the enzyme responsible for cleaving the polygalacturonan network (pectins) of the cell walls, is a candidate gene for controlling the MF/NMF flesh texture [9–11]. SH fruits barely exhibit softening on the tree or after harvest and remain crisp due to a lack of ethylene production [12–14]. *PpYUCCA11* (YUCCA Flavin mono-oxygenase gene), a candidate gene for the SH phenotype in peach, regulates auxin biosynthesis during peach fruit ripening [12, 15]. In addition to auxin and ethylene, abscisic acid (ABA) also plays critical roles in fruit ripening [16–18]. In peach, ABA appears to regulate ripening by affecting ethylene, cell wall and auxin-related genes, while ethylene acts as feedback regulator of ABA, contributing to rapid fruit softening [16, 19].

E3 proteins participate in numerous plant processes, including the light response [20], abiotic stress response [21, 22], flower and fruit development [23, 24], and hormone perception and signaling [25, 26]. In this study, the ubiquitin protein ligase genes from the peach genome database (https://www.rosaceae.org/) were identified. The chromosomal locations, gene duplication and phylogenetic analysis of each PpE3 were analyzed. The in situ-translated proteins were analyzed based on signature E3 domains. The transcriptome profiles of the PpE3 ligase genes from both a MF (‘Zhongyoutao 13’) and a SH (‘Zhongyoutao 16’) cultivar were examined during fruit ripening. Our aims were to identify what roles specific PpE3 ligases may play during ripening process of peach fruit.

## Results

### Identification and chromosomal distribution of E3 ubiquitin ligase genes in peach

In peach, 765 PpE3 ligase genes were identified through BLAST analysis using protein sequences from Arabidopsis and grape against the peach genome database (Additional file 1: Table S1). The PpE3 ligase genes account for almost 3.0% of the predicted proteins in the peach genome. The number of putative E3 ligase genes in peach was greater than identified in *V. vinifera* (677) but was significantly less than the numbers in six other species (Table 1).

**Table 1:** Number of E3 ubiquitin ligase genes, by subfamily, in peach and seven other plant species

	RING	F-box	BTB	U-box	SKP	Cullin	HECT	DDB^a^	RBX^a^	Total
Peach	338	267	67	54	16	13	7	3	NI	765
Arabidopsis^b^	465	654	79	62	21	10	7	5	2	1354
Grape^b^	330	153	63	56	10	8	9	NI	NI	677
Rice^b^	378	702	126	76	27	10	8	3	2	1387
Soybean^b^	725	418	77	120	18	24	19	3	2	1519
Poplar^b^	399	335	81	93	15	13	7	5	3	1027
Medicago^b^	294	539	41	38	36	12	9	NI	NI	1011
Maize^b^	401	325	129	119	31	6	21	NI	NI	1100

^a^Blast searches for RBX or DDB families have not found any qualified hits in some species (NI, none identified)

^b^The data were collected from the plantsUPS database (http://bioinformatics.cau.edu.cn) [2]

Normally there are nine subfamilies in the E3 ligase gene family. Only eight E3 subfamilies were identified in the peach genome, namely the BTB, Cullin, DDB, F-box, HECT, RING, SKP and U-box subfamilies. The RBX subfamily was not found in peach. The number of genes in the different subfamilies differed in peach. The largest number of genes, 338, was in the RING subfamily. The second largest was 267 in the F-box family. The RING and F-box subfamilies represented 79% of the predicted PpE3 ligase genes. The smallest subfamily was the DDB subfamily, with only three genes identified in peach.

For each of the PpE3 ligase genes, the exons, introns, additional domains and the length of each domain were analyzed (Additional file 1: Table S2). Most of PpE3 ligase genes contained introns, with numbers varying from 0 to 30. According to the number of introns, the PpE3 ligase genes were divided into 5 groups. Most of the PpE3 ligase genes, 408, contained between 1 and 5 introns. Of these genes, 191 genes lacked an intron in the second position. Only four PpE3 ligase genes, three in the RING and one in the HECT subfamily, have more than 20 introns. The number of introns in different subfamilies might have some relationship with their different functions.

All 765 of the identified PpE3 ligase genes were mapped onto one of the eight peach chromosomes (Additional file 2: Fig. S1). The largest number of genes (173) was located on chromosome 1, including 16 BTB, four Cullin, 78 F-box, two HECT, 61 RING, one SKP and 11 U-box genes. Only 68 genes were found on chromosome 8 (Additional file 1: Table S3). Members of the BTB, U-box, F-box and RING subfamilies could be found on every chromosome, while there was no Cullin gene on chromosomes 2, 6, or 7, no SKP gene on chromosomes 3, 5, or 6, no HECT gene on chromosomes 2, 3, or 5, and no DDB gene on chromosome 1, 3, 4, 5, or 8. The more abundant E3 ligase gene subfamilies were mainly present on the longer chromosomes (Chr 1 and Chr 6). This result indicated that the PpE3 ligase genes are not evenly distributed on each chromosome.

### Gene duplication pattern analysis

To explain the expansion and evolution of the E3 ligase gene family in peach, the patterns of gene duplication were analyzed and compared across the peach genome (Table 2). Dispersed gene duplication (DSD, 48% of 765) was responsible for the largest number of gene duplications among the PpE3 ligase genes, followed by tandem duplication (TD, 20%) and whole-genome duplication (WGD, 19%). However, the expansion of the PpE3 subfamilies did not all follow the same patterns. For the BTB subfamily, the largest number of gene pairs was derived from DSD (51% of 67), followed by WGD (21%), TD (16%) and proximal pairs (PD, 4%). DSD was also the most common duplication mode for the F-box (39% of 267), HECT (100% of 7), RING (53% of 338), SKP (44% of 16), and U-box (59% of 54) subfamilies. WGD was the second most common mode of duplication for the RING (27%), SKP (31%), U-box (30%) and Cullin (30%) subfamilies. In the F-box subfamily, TD (37% of 267) was the second most common mode of gene duplication. No duplicated gene pairs were detected in the DDB subfamily.

**Table 2:** The mode of duplication for the gene pairs in each E3 ligase subfamily in peach

Subfamily	Modes					Total
	DSD	TD	WGD	PD	Singleton	
BTB	34	11	14	3	5	67
Cullin	3	3	4	3		13
DDB					3	3
F-box	103	98	12	38	16	267
HECT	7					7
RING	179	34	92	15	15	338
SKP	7		5	4		16
U-box	32	5	16	0	1	54
Total	365	151	143	63	43	765

DSD: dispersed gene duplication; PD: proximal duplication; TD: tandem duplication; WGD: whole genome duplication. ‘null’ represents no finding of that gene duplication pattern within a subfamily

**Fig. 1:**
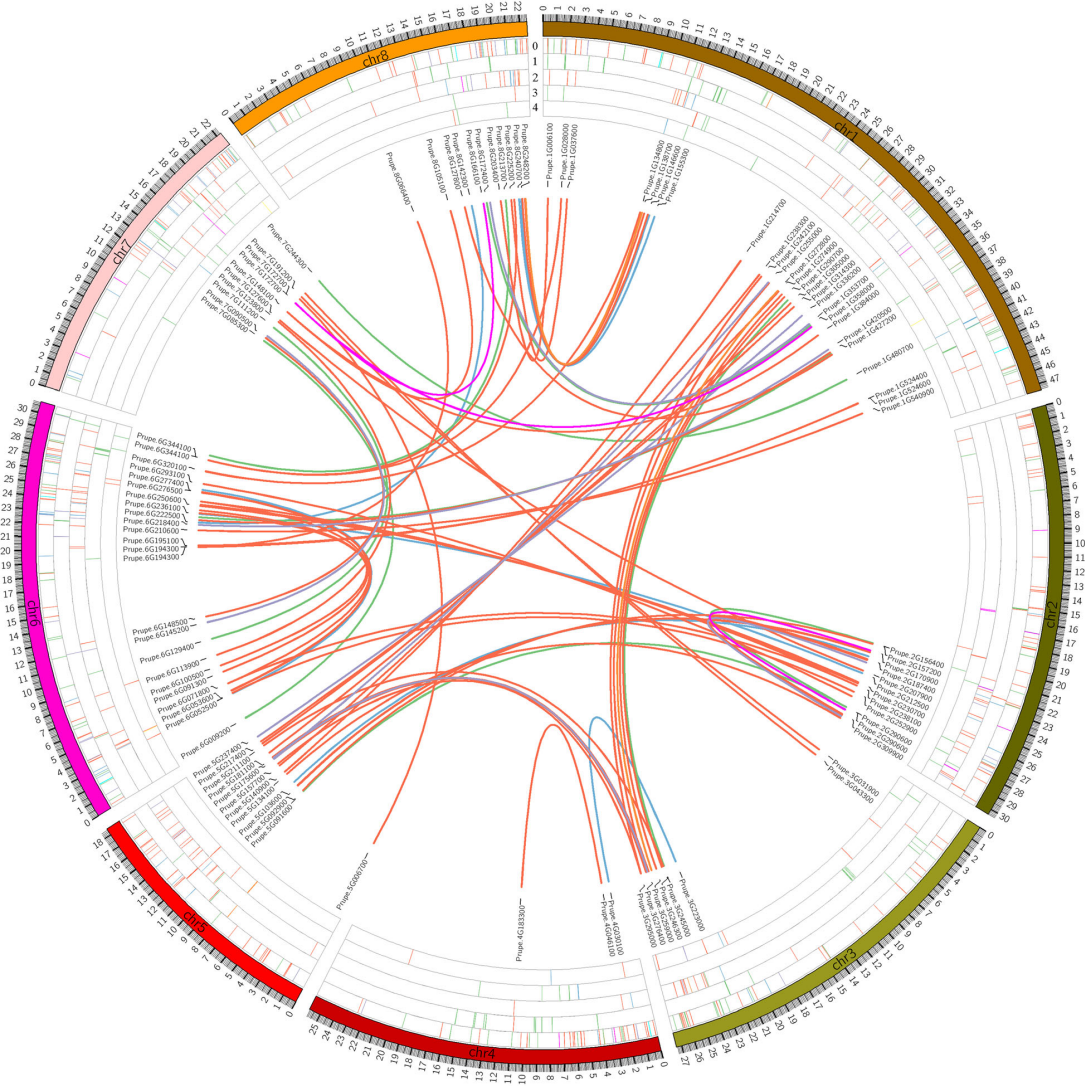
Genomic distribution and duplication of the PpE3 ligase genes across the 8 chromosomes of peach. The E3 genes are shown with the chromosome on which the occur. Syntenic pairs are linked with lines, with colors representing each subfamily: BTB, blue; Cullin, orange; DDB, yellow; F-box, green; HECT, cyan; RING, red; SKP magenta; and U-box, purple. Each concentric layer represents a type of gene duplication: Layer 0 represents DSD; Layer 1, tandem duplication (TD); Layer 2, whole-genome duplication (WGD); Layer 3, proximal pairs (PD); and Layer 4, singletons

The genomic distribution of the different types of gene duplications found in the PpE3 family was dissected (Fig. 1; Additional file 1: Table S4). The DSD, TD, WGD, PD and singleton patterns of each subfamily are shown in Fig. 1 as concentric layer 0, layer 1, layer 2, layer 3 and layer 4, respectively. Each syntenic pair is linked by a colored line, with the colors representing the different subfamilies. The DDB subfamily contained only singleton genes. DSD (layer 0) is the most prevalent gene duplication pattern in each subfamily and was found on each peach chromosome. The other patterns of gene duplication, including TD, WGD, PD and unduplicated singletons, in each subfamily occurred randomly among the different chromosomes. These results provide further insights into the expansion of the PpE3 family in peach.

### Classification and phylogenetic analysis

SMART and Pfam databases were used to detect the specific domains, shared domains, and other domains of the predicted PpE3 proteins in the eight subfamilies in peach. All E3 proteins in peach carried their subfamily-specific domain. In all, 81 other types of domains were identified in five of the E3 subfamilies (BTB, F-box, U-box, RING and HECT), some of which appeared in more than one subfamily. The classification of the BTB, F-box and U-box genes was based on the subfamily-specific domains plus additional domains.

**Fig. 2:**
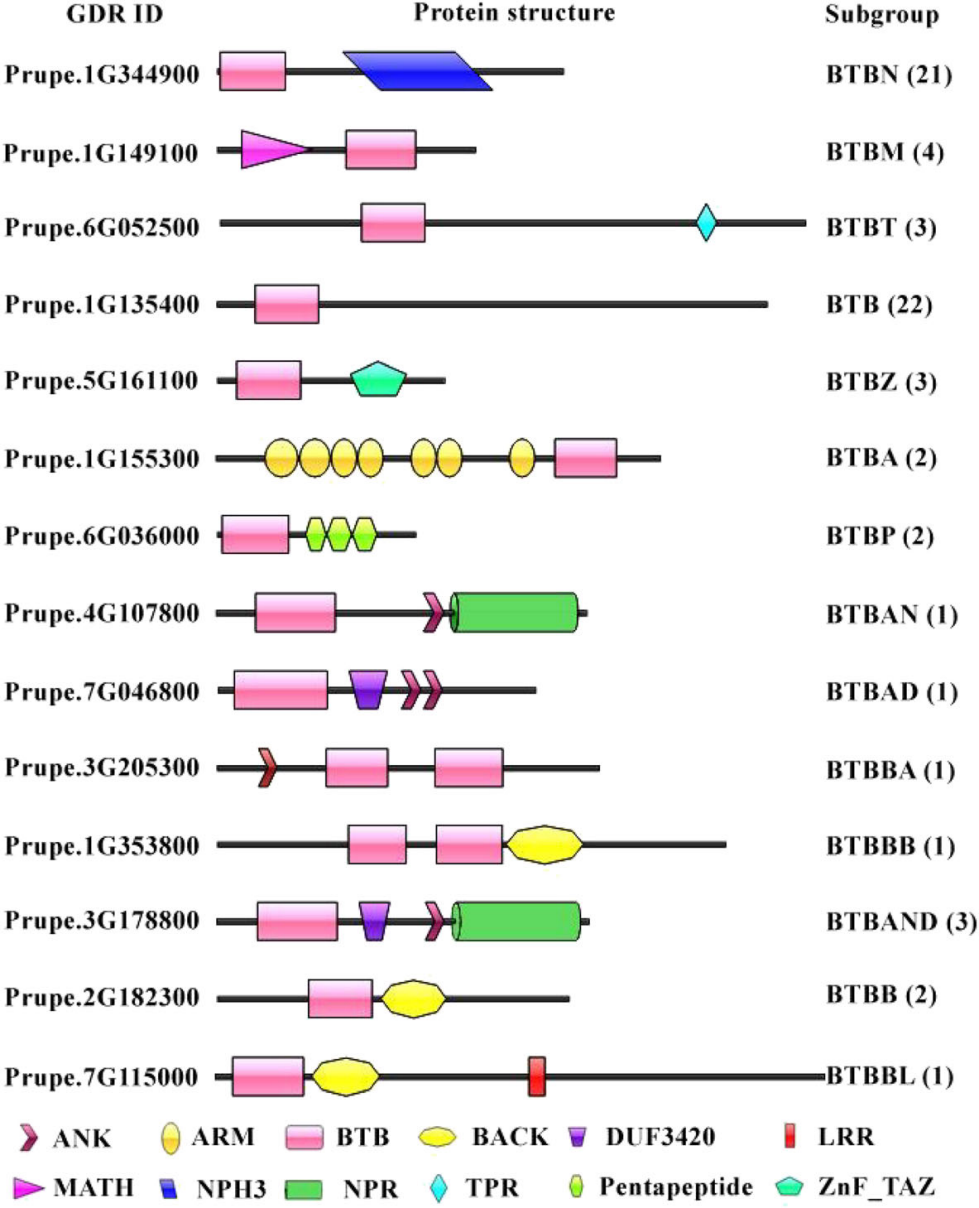
Predicted domains of BTB proteins representing each of the 14 subgroups. Members of the BTB subfamily with different domains are shown. The GDR protein IDs are shown on the left. The subgroup to which the representative BTB protein belongs and the number of members in each subgroup are shown on the right

**Fig. 3:**
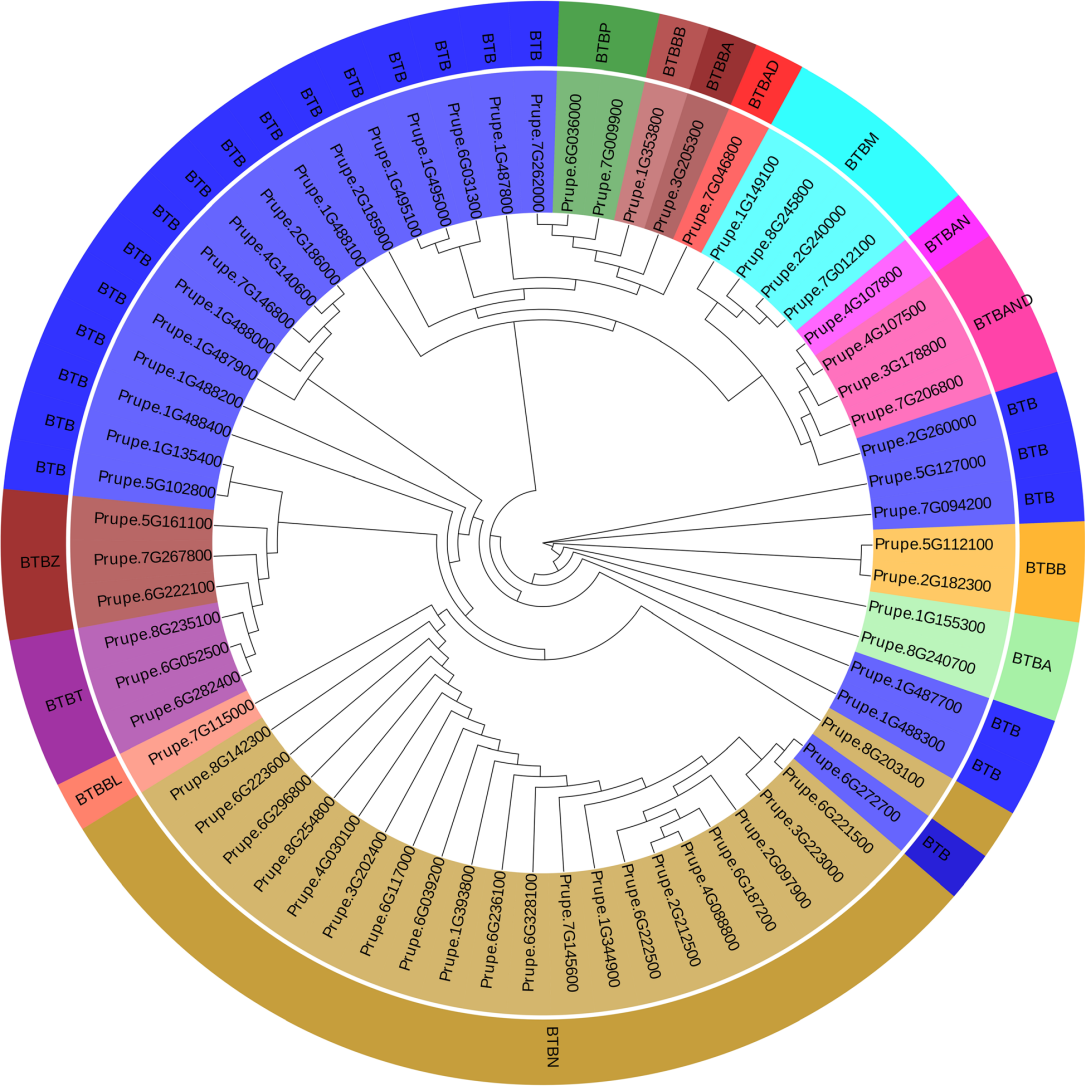
Phylogenetic analysis of the peach BTB subfamily. The tree was based on the alignment of the full-length BTB protein sequences. The phylogenetic tree was constructed by the MEGA6 program using the neighbor-joining method at 1000 bootstrap replicates

According to domains present in the BTB proteins, the BTB subfamily could be divided into 14 subgroups (Fig. 2), with 1 to 22 genes in each subgroup (Additional file 1: Table S5). Notably, 21 BTB genes containing the NPH3 domain were detected in BTBN subgroup. Four new subgroups of BTB proteins with different combinations of domains were identified in peach and named BTBAND (BTB-ANK-NPR-DUF), BTBBL (BTB-BACK-LRR), BTBP (BTB-Pentapeptide) and BTBAN (BTB-ANK-NPR). The observation of these new BTB subgroups in peach implies that these genes might play novel functions during the growth and development of peach. The phylogenetic analysis results of the BTB subfamily are shown in Fig. 3. Most subgroups were clustered together, such as the BTBN, BTBM and BTBT subgroups. The results were consistent using both the SMART and Pfam databases.

F-box proteins contain different domains and are classified into differing numbers of subgroups in different species. In peach, the FBX subgroup had 128 members with no other identified domains except the F-box motif. The other nine subgroups were named according to a previous study [27]. The numbers of genes in the different subgroups were quite different, with 64 in FBA, 19 in FBD, 2 in FBDL, 14 in FBL, 12 in FBK, 6 in FBP, 2 in FBW, 8 in FBDUF and 12 in FBO (Additional file 2: Fig. S2; Additional file 1: Table S5). Most members of some subgroups, such as FBX, FBA, FBP, FBK, FBL, and FBD, clustered together. Members of the FBO subgroup were scattered about the tree, possibly due to the non-uniform domains in the FBO subgroup (Additional file 2: Fig. S3; Additional file 1: Table S5).

The U-box proteins of peach were also divided into seven subgroups, according to the identity of the additional domain (Additional file 2: Figure S4; Additional file 1: Table S5). The U-box subgroup has 15 members that carry only the U-box domain without any other specific domains. Twenty-five U-box proteins contained the U-box domain and the ARMADILLO (ARM) domain, which was present in 1 to 6 repeats. Eight PpE3 proteins contained Pkinase domain. The TPR (TetratricoPeptide Repeat), UFD2 (Ubiquitin Fusion Degradation 2), and KAP (Kinesin-associated protein) subgroups each contained one member, and the WD40 subgroup had three members. In the phylogenetic tree of the U-box proteins, members of the ARM, U-box, Pkinase and WD40 subgroups clustered together (Additional file 2: Figure S5; Additional file 1: Table S5). The phylogenetic analysis of the eight subgroups partially supports our classification based on SMART and Pfam domains analyses.

There were 352 RING domains found in the 338 predicted proteins identified in the peach genome (Additional file 2: Figure S6; Additional file 1: Table S5). According to the spacing between the amino acids that bind the metal ligands or substitutions at one or more of the metal ligand positions, the RING subfamily was classified into six subgroups, including the RING-C2 (18), RING-G (3), RING-HC (198), RING-H2 (109), RING-S/T (4) and RING-v (20) subgroups. In the phylogenetic tree, members of most subgroups clustered together, with the exception of the proteins Prupe.1G303200 and Prupe.5G119800, two members of the RING-S/T subgroup that clustered with the RING-HC subgroup (Additional file 2: Fig. S7; Additional file 1: Table S5). This may be because these two genes evolved from the RING-HC subgroup.

The C-terminal end of the HECT proteins from peach contained an approximately 350-amino acid HECT domain (Additional file 1: Table S5). Proteins in the HECT subfamily also contained other domains, such as UBA (Ub-associated) and UIM (Ub-interacting), both of which are potentially important for ubiquitin ligase function. Based on the presence of additional protein motifs as predicted by the SMART and Pfam database, the HECT subfamily could be divided into three subgroups: (i) Subgroup I, only containing the HECT domain (4); (ii) Subgroup II, containing UBA, UIM and DUF domains (2); and (iii) Subgroup III, containing a UBQ (Ubiquitin homologues) domain (1) (Additional file 2: Figure S8; Additional file 1: Table S5). The phylogenetic tree of the HECT subfamily coincided with the classification results using the SMART and Pfam databases (Additional file 2: Figure S9; Additional file 1: Table S5).

### Expression of E3 ubiquitin ligase genes during fruit ripening in MF and SH peach

To reveal the expression patterns of PpE3 ligase genes in peach fruit, the transcriptome of fruit was analyzed during ripening in a MF and a SH cultivar across four stages of ripening. An average of 37,438,865 paired-end reads were obtained after filtering the reads of low quality and were mapped onto rRNA. About 95.5% of the high-quality reads were mapped against the peach reference genome (Additional file 1: Table S6).

**Fig. 4:**
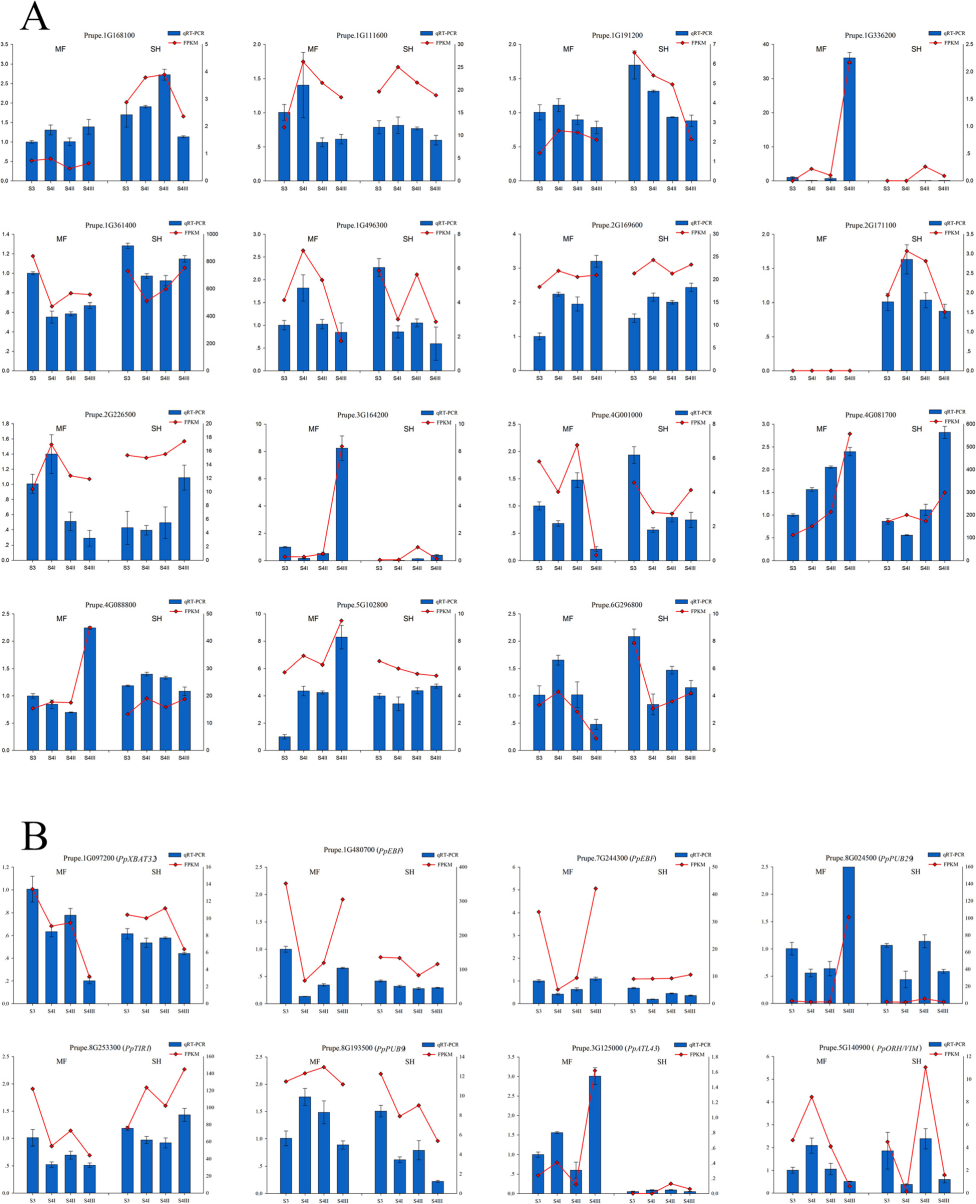
The relative gene expression of 23 PpE3 ligase genes was analyzed by qRT-PCR in MF and SH at four fruit ripening stages. **a** The relative gene expression of 15 randomly selected PpE3 genes; **b** The relative gene expression of eight genes related to ethylene, auxin and ABA signaling. The left y-axis indicates the relative gene expression; the right y-axis indicates the FPKM value

Among the 515 expressed PpE3 ligase genes, 231 differentially expressed genes (DEGs) were identified at the same-stage between the two peach cultivars (MF vs. SH) (Table 3; Additional file 1: Table S7). Fifteen randomly selected PpE3 ligase genes and eight genes related to ethylene, auxin and ABA pathway were used to confirm the expression levels by quantitative real-time PCR (qRT-PCR) during fruit ripening in the MF and SH cultivars. The qRT-PCR results were consistent with those of RNA-seq (Fig. 4). The number of DEGs in the different subfamilies was different (Table 3). The number of DEGs was highest for the RING subfamily, while the highest rate of differential expression within a subfamily was for the HECT subfamily.

**Table 3:** Number of expressed genes (EG) and differentially EGs (DEGs) of each subfamily during the peach fruit ripening process

	S3		S4I		S4II		S4III		DEGs at least in one stage
Subfamily	EG	DEGs	EG	DEGs	EG	DEGs	EG	DEGs	
RING (338)	242 (71.60%)	59 (17.46%)	244 (72.19%)	39 (11.54%)	246 (72.78%)	37 (10.95%)	241 (71.30%)	69 (20.41%)	119 (35.21%)
F-box (267)	122 (45.69%)	27 (10.11%)	127 (47.57%)	17 (6.37%)	124 (46.44%)	12 (4.49%)	124 (46.44%)	34 (12.73%)	53 (19.85%)
BTB (67)	51 (76.12%)	8 (11.94%)	50 (74.63%)	7 (10.45%)	54 (80.60%)	8 (11.94%)	49 (73.13%)	13 (19.40%)	24 (35.82%)
U-box (54)	37 (68.52%)	12 (22.22%)	40 (74.07%)	4 (7.41%)	39 (72.22%)	6 (11.11%)	39 (72.22%)	17 (31.48%)	23 (42.59%)
SKP (16)	8 (50.00%)	1 (6.25%)	8 (50.00%)	0 (0.00%)	8 (50.00%)	1 (6.26%)	8 (50.00%)	1 (6.26%)	3 (18.75%)
Cullin (13)	9 (69.23%)	5 (38.46%)	9 (69.23%)	1 (7.69%)	9 (69.23%)	1 (7.69%)	9 (69.23%)	0 (0.00%)	5 (38.46%)
HECT (7)	7 (100%)	1 (14.29%)	7 (100%)	0 (0.00%)	7 (100%)	0 (0.00%)	7 (100%)	2 (28.57%)	3 (42.86%)
DDB (3)	3 (100%)	0 (0.00%)	3 (100%)	0 (0.00%)	3 (100%)	0 (0.00%)	3 (100%)	1 (33.33%)	1 (33.33%)
Total (765)	479 (62.61%)	113 (14.77%)	488 (63.79%)	68 (8.89%)	490 (64.05%)	64 (8.37%)	480 (62.75%)	137 (17.91%)	231 (30.20%)

**Fig. 5:**
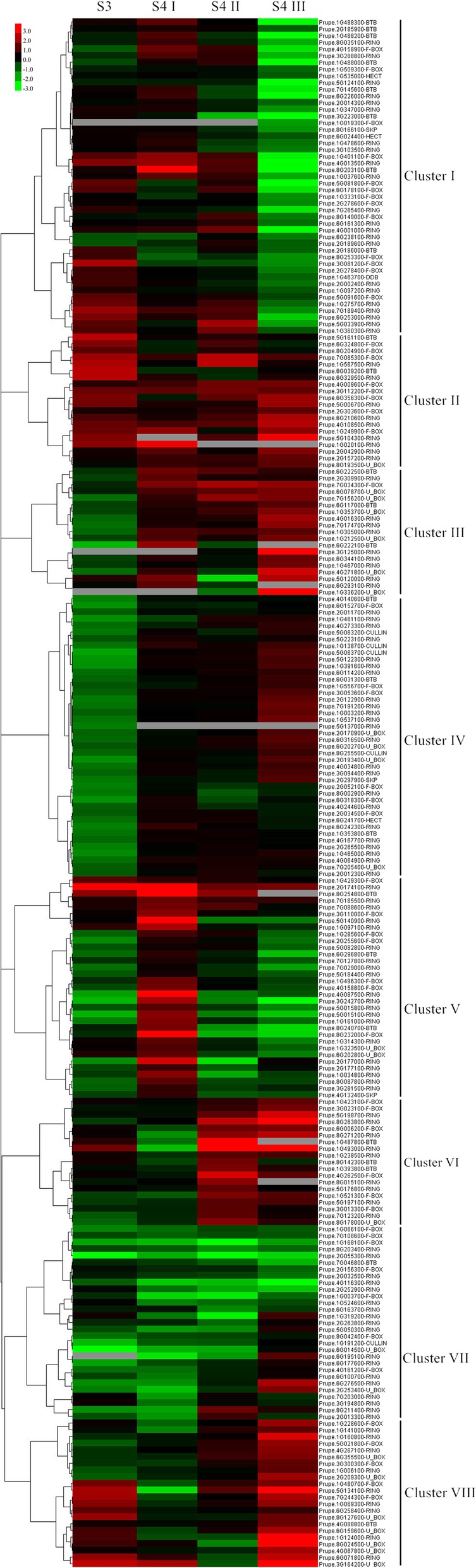
Expression profiles of PpE3 genes in peach fruits during ripening (stages S3-S4III) in MF and SH

According to their expression patterns at the same stage of ripening, the 231 DEGs could be classified into eight clusters (Fig. 5). In cluster I, 47 DEGs showed lower expression (1.7- to 42.0-fold) in MF at stage S4III. Twenty-two of the cluster I DEGs belonged to the RING subfamily. Surprisingly, the expression of Prupe.3G223000 was 42.0-fold lower in MF than in SH fruit. Prupe.3G223000 was annotated as a BTB/POZ protein with an NPH3 domain and has high homology with At3G22104 (function unknown). Meanwhile, one F-box gene, Prupe.8G253300 (PpTIR1), which is predicted to function as an auxin receptor TIR1 (Transport inhibitor response1), showed a 3.3-fold lower expression level in MF-S4III than in SH-S4III. Prupe.1G097200 (PpXBAT32), in the RING subfamily, has high homolog with AtXBAT32 (At5G57740).

DEGs in cluster II showed higher expression levels in MF peach at almost all analyzed stages of ripening. Twenty genes belonged to this cluster, including two BTB genes, eight F-box genes, nine RING genes and one U-box gene. Prupe.1G020100, a member of the RING subfamily, showed the greatest fold change between the MF and SH peaches (7.2-fold) at stage S4I. Another gene in this cluster, Prupe.8G193500 (PpPUB9), is a U-box gene with a high homology with AtPUB9 (At3G07360).

The transcript abundance of DEGs in cluster III was lower in MF than in SH at stage S3, but higher in MF at stage S4III. This cluster contained three BTB genes, one F-box gene, eight RING genes and six U-box genes. Prupe.6G222100, a BTB gene, showed the greatest fold change (− 4.6-fold in MF) at stage S3, but a higher expression level in MF at stage S4I. This gene (PpBT4) had high homology with ATBT4 (At5G67480, BTB and TAZ domain protein 4). Prupe.3G125000, a RING gene, showed the greatest fold change (27.0-fold in MF) at stage S4III. Prupe.3G125000 (PpATL43) has high homolog with ATL43 (At5G05810).

Cluster IV, the second largest group containing 42 genes, showed lower expression in MF at stage S3 but equivalent transcript levels in the other three stages. Their expression at stage 3 was 3.8- to 2.0-fold lower in MF than in SH. It was notable that four of the five CULLIN DEGs were in this cluster, Prupe.1G138700, Prupe.5G063200, Prupe.5G063700 and Prupe.8G255500. The CULLIN 1 homolog (Prupe.5G063700), which encodes a protein that forms a SCF complex with SKP1 and an F-box protein, showed the maximum fold change (− 3.8-fold) in this cluster.

The DEGs in cluster V, the third largest group with 33 genes, showed higher expression levels at stage S4I and lower expression levels at the other stages in the MF fruits. Prupe.5G140900, a RING gene, showed the highest fold change (64.9-fold in MF) at stage S4I but was 1.43-fold lower in MF at both stages S4II and S4III. Prupe.5G140900 had high homology with AtORTH/VIM (At1G57820, Orthrus/ Variant in methylation). Another RING gene, Prupe.3G242700, showed the greatest reduction in expression (− 8.1-fold) at stage S4III. The expression of the BTB gene Prupe.8G240700 showed the second lowest fold change (− 5.9-fold) in MF compared to SH at stage S4III.

Genes in cluster VI showed lower expression levels in MF at stages S3 and S4I, but higher expression levels at stages S4II and S4III. Prupe.1G493000 had the greatest swing in expression levels, with the greatest expression reduction (− 4.7-fold) at stage S4I and the greatest expression increase (21.1-fold) at stage S4II. Prupe.1G493000 (PpBB) is a RING protein highly homologous to AtBB (At3G63530, AtBIG BROTHER).

Cluster VII included 29 genes, most showing lower expression level in MF peaches at all four stages. Prupe.6G014500 showed the greatest fold change (− 26.0-fold) at stage S3 and is a U-box gene with high homology to AtPUB11 (At1G23030). However, the function of AtPUB11 is not clear yet.

Cluster VIII contained one BTB, five F-box, nine RING and seven U-box genes that had their highest expression levels in MF at stage S4III. The U-box gene Prupe.8G024500 (PpPUB29, highly homologous to MdPUB29, MDP0000928620) showed the greatest fold change (56-fold) in MF compared to SH at stage S4III. Among the five F-box genes in this cluster, two genes (Prupe.1G480700 and Prupe.7G244300) were annotated as EBFs (EIN3-binding factor).

## Discussion

### The number of E3 families and subfamilies varies greatly between different plant species

E3 ubiquitin ligases are encoded by numerous genes representing different subfamilies in the plant kingdom. The number of putative E3 ligase genes in peach was greater than the number identified in *V. vinifera* (677) but was significantly less than the numbers in six other species (Table 1). PpE3 ligase genes represented almost 3.0% of the predicted peach genes, higher than in rice, poplar, soybean, Medicago and maize (around 2.2%), but lower than in Arabidopsis (4.1%). This indicated that the number of E3 ligase genes in a genome is not associated with the size of the genome.

The number of genes in different subfamilies was also quite different (Table 1). The largest and second largest subfamilies, 338 in the RING and 267 in the F-box subfamilies, accounted for 79% of the peach E3 ligase genes. The same phenomenon was also seen in grape, maize, polar and soybean (Table 1) [2]. In eight different species, the F-box and RING finger E3 subfamilies are the two most abundant subfamily groups [2]. The number of genes in the DDB and RBX subfamilies was especially small (less than 10) in these eight species. Interestingly, the genes of abundant E3 gene subfamilies were mainly distributed on the longer chromosomes (Chr1 and Chr6), similar to the phenomenon in pear [27]. This indicated that the E3 ligase genes might be non-randomly distributed on the chromosomes of peach.

The exon-intron structure of 354 duplicated F-box genes were analyzed in *A. thaliana*. There were 137 (38.7%) F-box genes that carried a different number of exons, while 211 (59.6%) had the same numbers of exons, although the homologous exons were of different length of exons [28]. Duplication of genes allows genetic drift, that includes both alteration of exon-intron structure and the coded amino acids [28]. In this study, the exon-intron number differed in each subfamily. The analysis of exon-intron number might provide more information on the mechanism behind evolution of exon-intron structure and distinct biochemical functions.

### Different modes of gene duplication occurred within each subfamily

There are several patterns of gene duplication, with whole-genome duplication (WGD), tandem duplication (TD), dispersed gene duplication (DSD) and proximal duplication (PD) being the primary contributors to the expansion of specific gene families in plant genomes [27, 29, 30]. The gene duplication within the different PpE3 subfamilies (Fig. 1, Table 2) showed patterns that were inconsistent with those of other plant species [27, 31–33]. The F-box gene family was expanded in some species through TD and SD [31, 32] and in pear through WGD and DSD [27]. In peach, the data indicated that the F-box family was expanded more through DSD and TD. For the RING gene family in apple, TD and WGD were the major contributors of expansion [33]. In peach, the expansion of the RING subfamily was mainly through DSD and WGD. The same phenomenon was also observed for the BTB and U-Box subfamilies. Based on the above results, DSD is the primary means of gene duplication for the F-box, U-box, RING, BTB and SKP subfamilies in peach. It is most likely that peach has not undergone a recent whole-genome duplication [34].

### Classification and domain analysis in F-box, BTB, U-box, RING and HECT subfamilies

BTB gene family has been discovered in animals and plants [26, 35, 36]. In this study, four new subgroups of BTBs, namely BTBAND, BTBBL, BTBP and BTBAN, were firstly identified in peach. Domains within these new of BTB subgroups were detected in previous studies [26, 35, 36], but the combinations of the domains are novel. The observation of new subgroups of BTB genes in peach might imply that these genes play new or unknown functions in the growth and development of peach. For instance, the new subgroup BTBAND was a combination of ANK, NPR and DUF domains. It has been documented that ANK (Ankyrin) domains mediate diverse protein-protein interactions [37]. In rice, ANK-containing proteins participated in various physiological processes, such as responses to NAA (1-naphthylacetic acid) and light treatments [38]. NPR1 (Non-expressor of pathogenesis-related (PR) genes 1) is a master immune regulator [39]. DUF (Domain of unknown function) domains have no characterized function [40]. The new subgroup BTBAND may function by integrating the roles of BTB, ANK, NPR1 and DUF domains. Among the PpE3 ligase genes, the NPH3 (Non-phototropic hypocotyl 3) domain was only observed in the BTB subfamily. During the phototropic response, NPH3 interacts with and mediates the ubiquitination of PHOT1 (Phototropic 1), leading to auxin-regulated differential growth [41]. NPH3 also seems to be specific to higher plants [42, 43]. We speculated that these BTB genes may play a part in phototropic responses blue light.

The F-box proteins in Arabidopsis, pear and rice have been grouped into 15, 12 and 10 subgroups, respectively [20, 27, 29]. In general, additional domains at the C-terminal of an F-box protein recruit specific substrates to the SCF complex and may indicate functional diversification [44]. In peach, 13 F-box proteins were predicted to contain Kelch domains. Genes encoding F-box proteins containing a Kelch domain negatively regulate flavonoid accumulation in rice [45], and in melon [46].

In peach, the U-box proteins were divided into seven subgroups, but the U-box proteins of soybean were grouped into eight subgroups [47]. One PpE3 protein was predicted to contain a TPR domain. The TPR domain interacts with other proteins to form complexes [48]. The TPR1 and TPR2A domains of Hop could specifically recognize the C-terminal heptapeptides of HSP70 and HSP90, respectively [49]. SGT1, which contains a TPR domain and acts as a co-chaperone between HSP90 and SKP1, was proposed to regulate the assembly of SCF complexes [50, 51].

### The PpE3 ubiquitin ligase genes exhibit diverse expression patterns during fruit ripening in peach

The shelf life of peach fruit is greatly affected by the rate of flesh softening, which is a complex process. The molecular and genetic mechanisms regulating flesh softening are unclear. In this study, the transcriptome profiles of the predicted PpE3 ligase genes in MF and SH peaches were compared during fruit ripening. Of the identified 765 PpE3 ligase genes, 515 (67.32%) were expressed in fruit flesh, and 231 (30.20%) were differentially expressed. These DEGs showed different expression levels in the two cultivars when comparing the same stage of ripening, indicating that these E3 ligase genes may be involved in peach fruit softening. Among the eight E3 ligase subfamilies, the RING and F-box subfamilies had greater numbers of DEGs in peach fruit.

There were several DEGs similar to E3 genes known to be involved in ethylene biosynthesis or signal transduction, such as *PpXBAT32*, *PpEBF1s* and *PpPUB29*. In Arabidopsis, the RING-type E3 ligase XBAT32 maintains proper levels of ethylene by targeting ACS4 (1-aminocyclopropane-1-carboxylate synthase 4) and ACS7 for proteasomal degradation [52]. Overexpression of *AtEBF1* brings about ethylene insensitivity, which indicates the inverse correlation between EIN3 and the F-box proteins [53]. PpPUB29 is homologous with MdPUB29, which directly ubiquitinates the MdbHLH3 protein, which is related to the regulation of ethylene biosynthesis. MdPUB29 is inhibited by high glucose in apple (*Malus domestica*) [54]. In this study, the expression level of *PpPUB29* was much higher in MF than in SH at stage S4III. We speculated that PpPUB29 may influence ethylene production in MF.

Auxin regulates many aspects of plant growth and development, such as elongation and fruit development [12, 55]. Some PpE3s with homology to genes regulated by auxin showed differentially expression in the two types of peach fruit, such as *PpBT4* and *PpBB*. In Arabidopsis, the five members of the BTBZ subgroup (AtBT1-AtBT5) showed functional redundancy and transcriptional compensation among the subgroup members and played vital roles in multiple responses and plant development [56–58]. The expression levels of *AtBT1*, *2*, *4*, and *5* were enhanced by auxin [56]. The expression of *PpBT4* might be regulated by IAA in MF peach. IAA levels increased in MF peach [59], while it did not increase in SH peach during fruit ripening [12, 60]. In Arabidopsis, AtBB performs a central function in controlling organ size by degrading critical growth stimulators in a dosage-dependent manner [61]. Given the effects of phytohormones on organ growth [62], the higher expression levels of *PpBB* in MF at stages S4II and S4III might also be due to higher IAA levels in MF. Some components of the ethylene-response machinery are used by auxin signaling to drive ripening [12, 15, 60]. It implied that the functions of AtBB and PpBB might be different in Arabidopsis and peach. We also found that a few DEGs were predicted to function in auxin signal transduction, like *TIR1*, which is an auxin receptor. In this study, *PpTIR1* showed significantly lower expression (3.3-fold) in MF peach compared to in SH peach at S4III.

Some other DEGs have homologs with known functions that could be related to fruit ripening and softening. A few of E3 ligase genes are homologous to genes involved in ABA signal transduction pathway, such as the RING gene *AtATL43*, the U-box gene *AtPUB9*, and the BTB gene *AtCRL3*, which mediate ubiquitination of ABA receptors [5]. In this study, *PpATL43* and *PpPUB9* showed higher expression in MF peach at stage S4III. AtATL43 plays a positive role and AtPUB9 a negative role in ABA signaling [63, 64]. This contrast implies that the signal transduction of ABA might be in a state of dynamic balance. The gene *PpORTH/VIM* showed much higher expression in MF compared to SH peach at S4I. The Arabidopsis *AtORTH/VIM* gene encodes a protein that regulates DNA methylation through it ubiquitin E3 ligase function [65]. These findings were consistent with previous reports that fruit ripening and softening are related to ethylene, IAA, ABA, and DNA methylation [11, 12, 19, 66].

## Conclusion

In this study, 765 putative E3 ubiquitin ligase genes were identified in the peach genome, which could be divided into eight subfamilies according to the characterization of known functional domains. The RBX subfamily was not detected in peach. The primary mode of gene duplication of the PpE3 ligase genes was dispersed gene duplication (DSD). Four new subgroups of the BTB family were first identified in this study. Among the 515 *PpE3* ligase genes expressed during fruit ripening, 231 genes were differentially expressed in MF and SH peach varieties at the same stage of fruit ripening. All of the DEGs showed a variety of expression patterns during fruit ripening. Many genes were directly or indirectly related to auxin, ethylene, or ABA biosynthesis or signal transduction and DNA methylation, such as *PpXBAT32*, *PpEBF1s*, *PpPUB29*, *PpTIR1*, *PpPUB9*, *PpATL43* and *PpORTH/VIM*. These results inspire us to further explore the roles of these E3 ubiquitin ligase genes in peach fruit ripening and softening to discover the gene regulatory network of this process.

## Methods

### Plant materials and RNA-seq

The two peach (*Prunus persica* (L.) Batsch) cultivars used in this study have different flesh textures, one a Melting Flesh cultivar named ‘Zhongyoutao 13’ (MF) and one a Stony Hard cultivar named ‘Zhongyoutao 16’ (SH). Trees of the two cultivars were planted in an orchard at the Institute of Zhengzhou Fruit Research, Chinese Academy of Agriculture Science (Zhengzhou, China) and were 6-years-old at the time of harvest. Peach fruit were collected at pre-ripening (S3) and ripening (S4 I, S4 II, and S4 III) according to Tonutti et al. [67]. The MF (Zhongyoutao 13) fruits were collected at 87 (S3), 90 (S4 I), 93 (S4 II) and 96 (S4 III) days after full bloom (DAFB). The SH (Zhongyoutao 16) fruits were collected at 79 (S3), 83 (S4 I), 87 (S4 II) and 90 (S4 III) DAFB. Twenty fruits were gathered from five self-pollinated trees at each stage. The fruit mesocarp was separated, immediately frozen in liquid nitrogen, and stored at − 80 °C for RNA-seq. Eight libraries were generated from the samples of the two peach cultivars at the four stages (S3-S4III) using an Illumina kit and were sequenced by an Illumina HiSeq™2500 sequencer.

### Identification of candidate genes and chromosomal distribution analysis

The deduced protein sequences of the E3 ubiquitin ligase genes from Arabidopsis and grape were downloaded from the NCBI (http://www.ncbi.nlm.nih.gov). The peach genome dataset was downloaded from the Genome Database for Rosaceae (GDR; https://www.rosaceae.org/). Different subfamilies of E3 ubiquitin ligase proteins from Arabidopsis and grape were used as queries to search against the whole peach genome by the BLASTP (http://blast.ncbi.nlm.nih.gov/) program with an E-value <1e-6. The retrieved sequences were used for domain searches in the Pfam (http://pfam.jouy.inra.fr/) and SMART (http://smart.embl-heidelberg.de/) databases with an E-value cutoff level of 1.0. Candidate genes lacking indispensable E3 domains were removed.

The ubiquitin ligase genes were anchored to peach chromosomes based on the location information extracted from the genome annotation ‘*Prunus_persica*_ v2.0.a1.gene.gff3’ downloaded from the GDR. The physical chromosomal locations and syntenic genes were drafted by Circos software.

The numbers of ubiquitin ligase genes in other plant species were obtained from plantsUPS (a database of the Ubiquitin Proteasome System of plants) [2].

### Synteny analysis and dissection of different modes of gene duplication

The analysis of gene synteny in the peach genome was performed using the Plant Genomic Duplication Database (http:// chibba.agtec.uga.edu/duplication/) [68]. To identify homologous gene pairs, BLASTP was carried out (E-value <1e-5). Gene duplication modes were identified using MCScanX [69].

### Classification and phylogenetic analysis

The potential domains contained in each BTB, F-box, HECT, RING, U-box, Cullin, SKP and DDB sequence were detected by the SMART and Pfam web servers. According to additional domains found in the BTB, F-box and U-box proteins, these genes were grouped into different subgroups. Based on the amino acid residues in conserved positions and the predicted distances between metal ligands, the RING-type for each peach RING domain was determined.

Phylogenetic trees were constructed for the BTB, F-box, HECT, RING and U-box subfamilies using each full-length protein sequence and the MEGA6 program according to the Neighbor-Joining (NJ) method, with 1000 bootstrap replicates [70].

### Expression analysis

The RNA-seq data of 125-bp paired-ends reads were analyzed to explore the expression patterns of PpE3 ligase genes in peach fruit. The RNA-seq data have been deposited in NCBI under accession number (PRJNA398309). The filtered reads of high quality and no rRNA were mapped to the peach reference genome (https://www.rosaceae.org/species/prunus_persica/genome_v2.0.a1) using TopHat2 [71]. Gene expression levels were measured and expressed in fragments per kilobase of exon per million fragments mapped (FPKM) [72]. Genes with FPKM values > 1 were defined as showing significant expression. Between MF and SH at the same stage, genes were considered to show differential expression at log_2_ > 2 and were identified for further analysis. The heatmap of the expression patterns of PpE3 ligase genes was generated by Cluster 3.0 software.

To confirm the differential expression of PpE3 ligase genes identified by RNA-seq, 15 randomly selected PpE3 ligase genes and eight PpE3 ligase genes with known functions in other plants were analyzed by quantitative real-time PCR (qRT-PCR) at the four stages of ripening in ‘Zhongyoutao 13’ and ‘Zhongyoutao 16’. Total RNA was isolated from peach fruit using the Plant Total RNA Isolation Kit (Sangon, Shanghai, China). The quality and concentration of RNA were detected by 1% agarose gels and NanoDrop ND-2000 spectrophotometer, respectively. The cDNA was synthesized from RNA with PrimeScript™ RT reagent Kit (TaKaRa, Japan), and then diluted for qRT-PCR. qRT-PCR data were normalized using peach *RPL13* (Ribosomal Protein L13) gene. The qRT-PCR was performed on a QuantStudio5 system platform (Thremo Lifetech ABI). All primer sequences used in this study are listed in Additional file 1: Table S8. Three biological replicates of each sample were used for qRT-PCR analysis.

## Supplementary information

**Additional file 1: Table S1.** The characteristics of E3 ligase genes in peach. (XLSX 97 kb)**. Table S2.** The distribution of introns among different PpE3s. (XLSX 10 kb)**. Table S3.** Chromosomal distribution of different PpE3 subfamilies in peach. (XLSX 11 kb)**. Table S4.** Prevalence of duplication modes in the PpE3 subfamilies. (XLSX 40 kb)**. Table S5.** Domain structure of predicted PpE3 ligase proteins in different subfamilies. (XLSX 108 kb)**. Table S6.** The statistical analysis of data by transcriptome sequencing (XLSX 14 kb)**. Table S7.** Fold changes in PpE3 ligase gene expression between MF and SH peach fruits at the same stage of ripening. (XLSX 96 kb)**. Table S8.** Primers used in qRT-PCR. (XLSX 14 kb).

**Additional file 2: Figure S1.** Chromosomal distribution of different PpE3 subfamilies in peach. (JPG 321 Kb). **Figure S2.** Predicted domains of F-box proteins representing each subgroup. (JPG 1.3 MB). **Figure S3.** Phylogenetic analysis of the peach F-box subfamily. (JPG 8.1 MB). **Figure S4.** Predicted domains of U-box proteins representing each subgroup. (JPG 2.0 MB). **Figure S5.** Phylogenetic analysis of the peach U-box subfamily. (JPG 3.7 MB). **Figure S6.** Sequence logo of the overrepresented motifs found in the RING-C2, RING-H2, RING-HC, RING-G, RING-v or RING-S/T domains of the RING proteins predicted from the peach genome. (JPG 9.2 MB). **Figure S7.** Phylogenetic analysis of the peach RING subfamily. (JPG 8.6 MB). **Figure S8.** Predicted domains of HECT proteins representing each subgroup. (JPG 631.3 kb). **Figure S9.** Prehylogenetic analysis of the peach HECT subfamily. (JPG 920.5 kb).

## Data Availability

All data presented in this study are provided either in the manuscript or additional files. Raw reads data has been deposited in NCBI under accession number PRJNA398309.

## Abbreviations

ABA: Abscisic Acid
ACS: 1-aminocyclopropane-1- carboxylate synthases
ANK: Ankyrin
ARM: Armadillo
BTB: Broad-complex, tramtrack and bric-a-brac
DAFB: Days after full bloom
DDB: Damaged DNA binding protein
DEG: Differentially expressed gene
DSD: Dispersed gene duplication
DUF: Domain of unknown function
EBF: EIN3-binding factor
FPKM: Fragments per kilobase of exon per million fragments mapped
GDR: Genome database for Rosaceae
HECT: Homologous to the E6-associated protein carboxyl terminus
KAP: Kinesin-associated protein
MF: Melting flesh
NAA: 1-naphthylacetic acid
NCBI: National Center for Biotechnology Information
NJ: Neighbor-Joining
NMF: Non-melting flesh
NPH3: Non-phototropic hypocotyl 3
NPR1: Non-expressor of pathogenesis-related (PR) genes 1
ORTH/VIM: Orthrus/Variant in methylation
PD: Proximal pairs
PHOT1: Phototropic 1
PUB: Plant U-box
qRT-PCR: Quantitative Real-Time PCR
RING: Really interesting new gene
SCF: SKP-Cullin-F-box
SH: Stony hard
TD: Tandem duplication
TIR1: Transport inhibitor response 1
TPR: Tetratrico peptide repeat
UBA: Ub-associated
UFD2: Ubiquitin fusion degradation 2
UGT: UDP-glycosyltransferases
UIM: Ub-interacting motif
WGD: Whole-genome duplication
XBAT32: XB3 ortholog 2 in *Arabidopsis thaliana*
