# Supplementary figures and images for: Genome-wide identification and transcriptome profiling reveal that E3 ubiquitin ligase genes relevant to ethylene, auxin and abscisic acid are differentially expressed in the fruits of melting flesh and stony hard peach varieties
Source: BMC Genomics. 2019 Nov 21;20:892. doi: 10.1186/s12864-019-6258-0 (PMC6873611; doi:10.1186/s12864-019-6258-0)

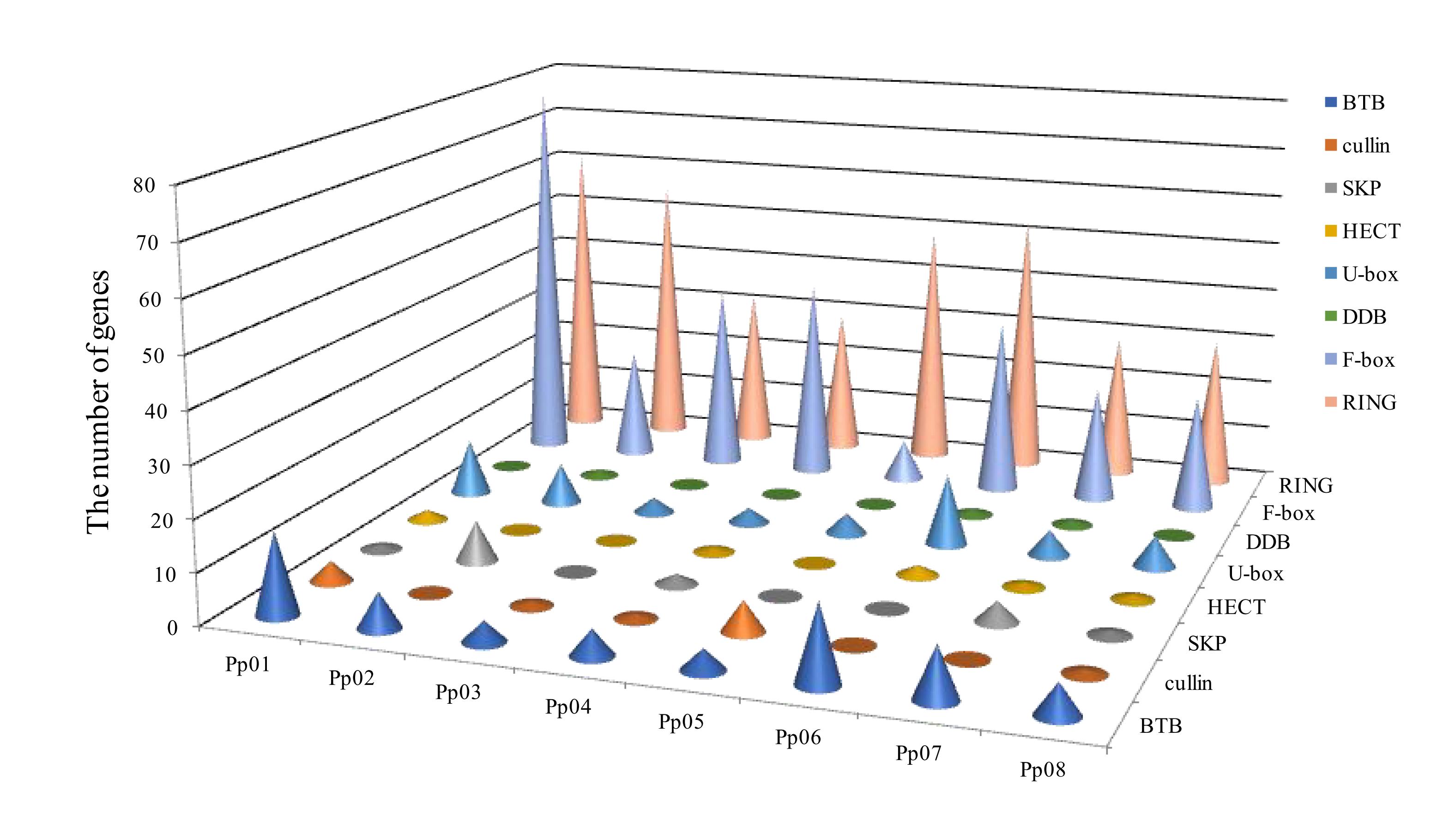

Supplement: Supplementary file 2 — Additional file 2: Figure S1. Chromosomal distribution of different PpE3 subfamilies in peach. (JPG 321 Kb). Figure S2. Predicted domains of F-box proteins representing each subgroup. (JPG 1.3 MB). Figure S3. Phylogenetic analysis of the peach F-box subfamily. (JPG 8.1 MB). Figure S4. Predicted domains of U-box proteins representing each subgroup. (JPG 2.0 MB). Figure S5. Phylogenetic analysis of the peach U-box subfamily. (JPG 3.7 MB). Figure S6. Sequence logo of the overrepresented motifs found in the RING-C2, RING-H2, RING-HC, RING-G, RING-v or RING-S/T domains of the RING proteins predicted from the peach genome. (JPG 9.2 MB). Figure S7. Phylogenetic analysis of the peach RING subfamily. (JPG 8.6 MB). Figure S8. Predicted domains of HECT proteins representing each subgroup. (JPG 631.3 kb). Figure S9. Prehylogenetic analysis of the peach HECT subfamily. (JPG 920.5 kb). [file 12864_2019_6258_MOESM2_ESM.zip › Additional file 2 Figure S1.jpg]

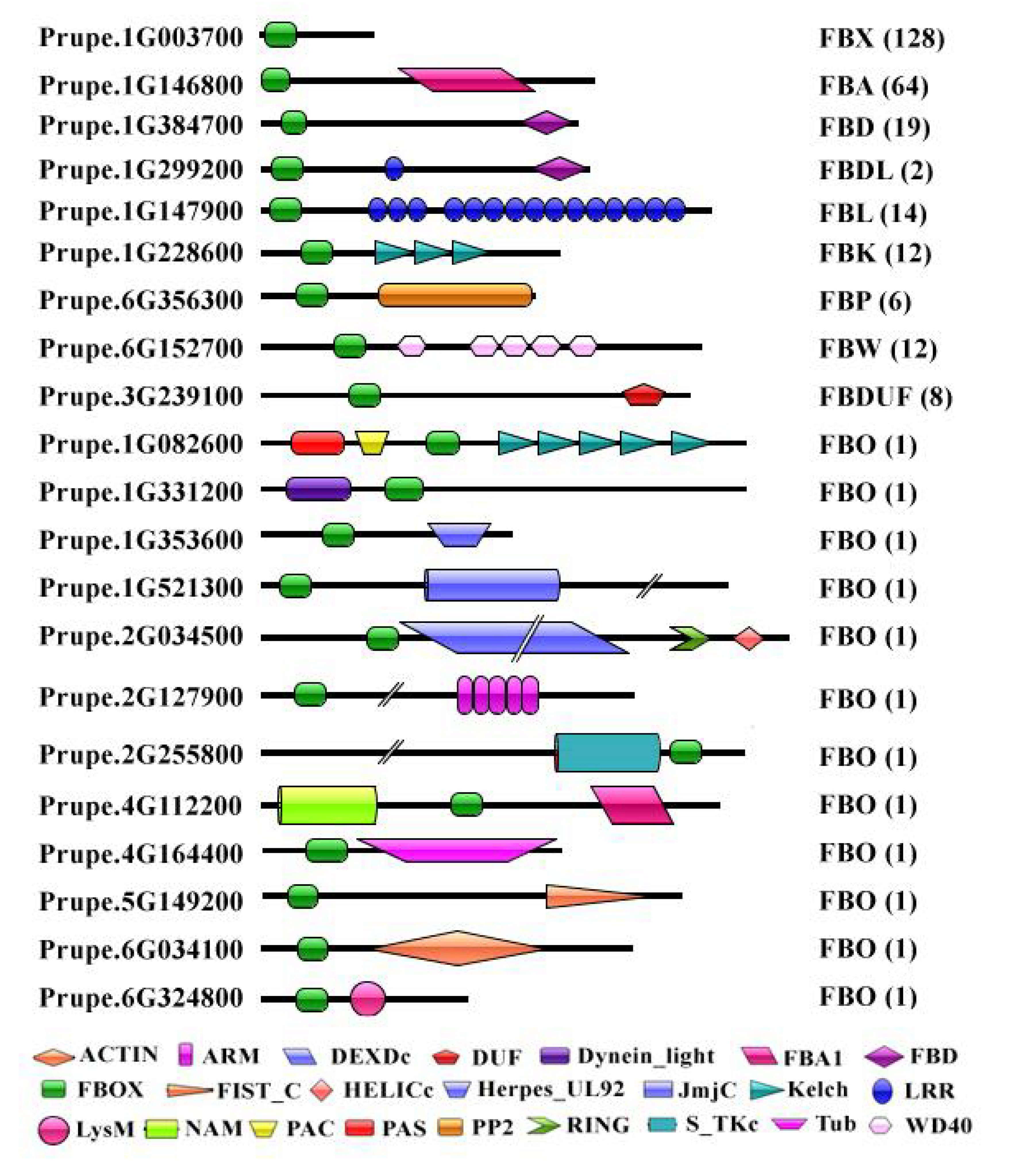

Supplement: Supplementary file 2 — Additional file 2: Figure S1. Chromosomal distribution of different PpE3 subfamilies in peach. (JPG 321 Kb). Figure S2. Predicted domains of F-box proteins representing each subgroup. (JPG 1.3 MB). Figure S3. Phylogenetic analysis of the peach F-box subfamily. (JPG 8.1 MB). Figure S4. Predicted domains of U-box proteins representing each subgroup. (JPG 2.0 MB). Figure S5. Phylogenetic analysis of the peach U-box subfamily. (JPG 3.7 MB). Figure S6. Sequence logo of the overrepresented motifs found in the RING-C2, RING-H2, RING-HC, RING-G, RING-v or RING-S/T domains of the RING proteins predicted from the peach genome. (JPG 9.2 MB). Figure S7. Phylogenetic analysis of the peach RING subfamily. (JPG 8.6 MB). Figure S8. Predicted domains of HECT proteins representing each subgroup. (JPG 631.3 kb). Figure S9. Prehylogenetic analysis of the peach HECT subfamily. (JPG 920.5 kb). [file 12864_2019_6258_MOESM2_ESM.zip › Additional file 2 Figure S2.jpg]

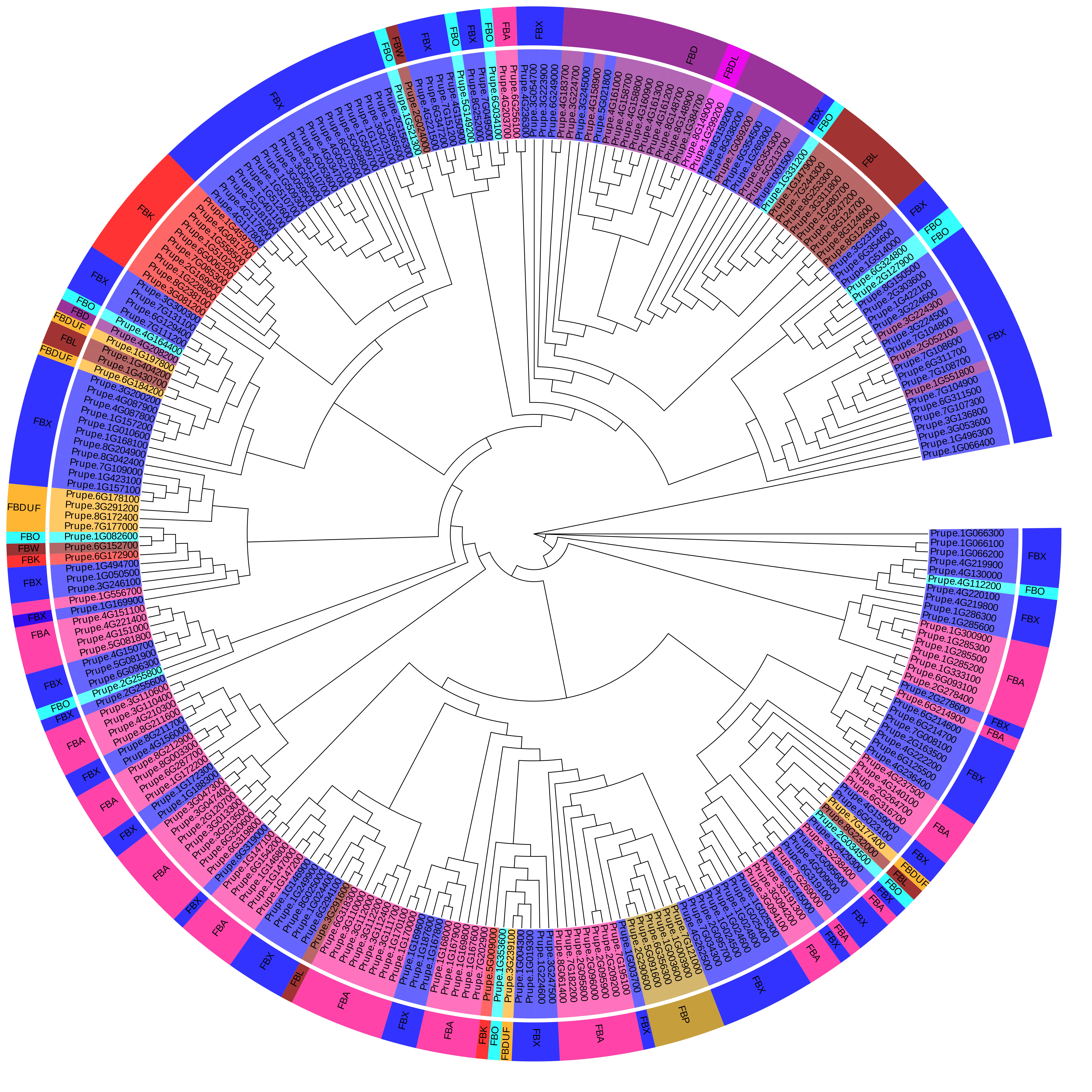

Supplement: Supplementary file 2 — Additional file 2: Figure S1. Chromosomal distribution of different PpE3 subfamilies in peach. (JPG 321 Kb). Figure S2. Predicted domains of F-box proteins representing each subgroup. (JPG 1.3 MB). Figure S3. Phylogenetic analysis of the peach F-box subfamily. (JPG 8.1 MB). Figure S4. Predicted domains of U-box proteins representing each subgroup. (JPG 2.0 MB). Figure S5. Phylogenetic analysis of the peach U-box subfamily. (JPG 3.7 MB). Figure S6. Sequence logo of the overrepresented motifs found in the RING-C2, RING-H2, RING-HC, RING-G, RING-v or RING-S/T domains of the RING proteins predicted from the peach genome. (JPG 9.2 MB). Figure S7. Phylogenetic analysis of the peach RING subfamily. (JPG 8.6 MB). Figure S8. Predicted domains of HECT proteins representing each subgroup. (JPG 631.3 kb). Figure S9. Prehylogenetic analysis of the peach HECT subfamily. (JPG 920.5 kb). [file 12864_2019_6258_MOESM2_ESM.zip › Additional file 2 Figure S3.jpg]

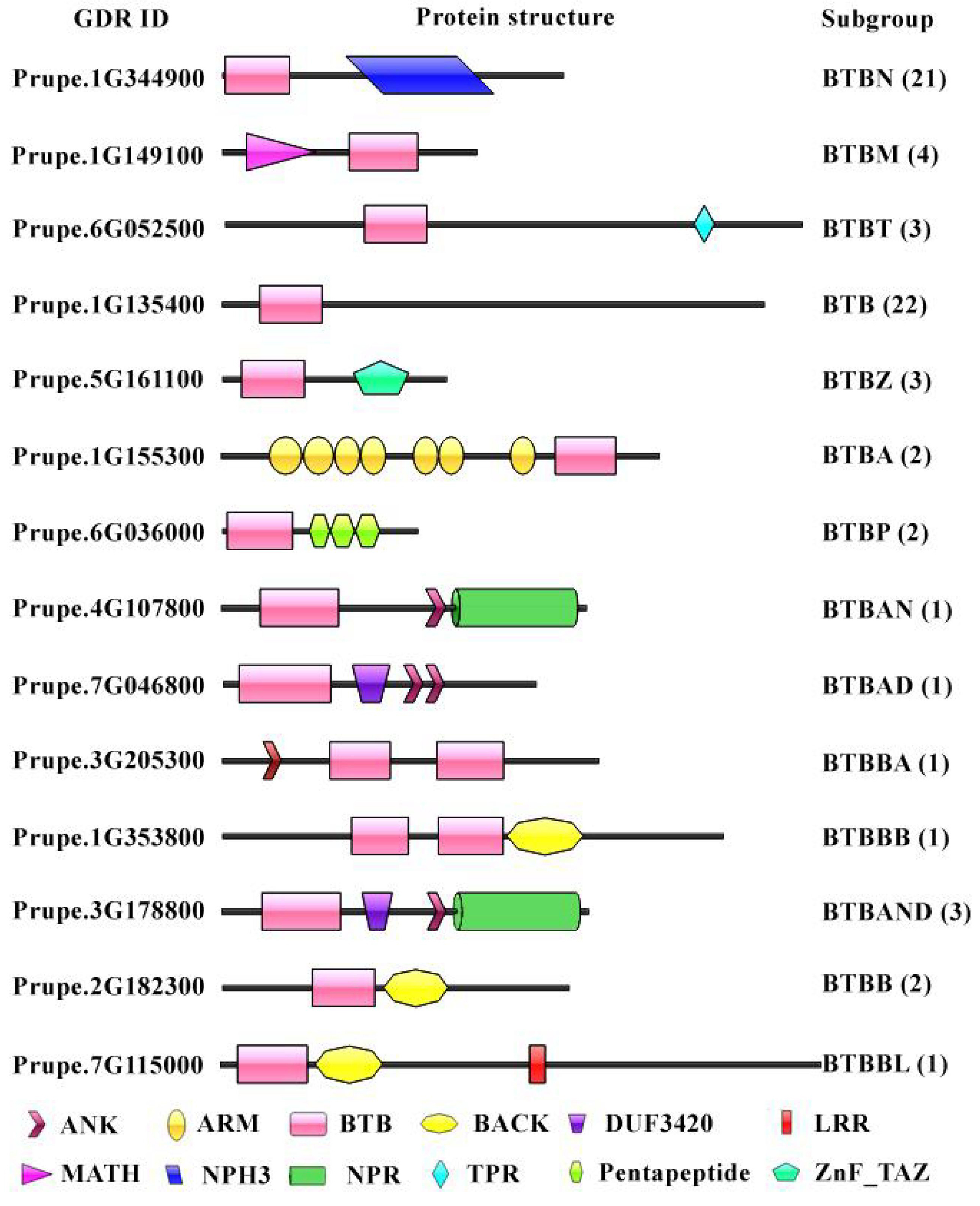

Supplement: Supplementary file 2 — Additional file 2: Figure S1. Chromosomal distribution of different PpE3 subfamilies in peach. (JPG 321 Kb). Figure S2. Predicted domains of F-box proteins representing each subgroup. (JPG 1.3 MB). Figure S3. Phylogenetic analysis of the peach F-box subfamily. (JPG 8.1 MB). Figure S4. Predicted domains of U-box proteins representing each subgroup. (JPG 2.0 MB). Figure S5. Phylogenetic analysis of the peach U-box subfamily. (JPG 3.7 MB). Figure S6. Sequence logo of the overrepresented motifs found in the RING-C2, RING-H2, RING-HC, RING-G, RING-v or RING-S/T domains of the RING proteins predicted from the peach genome. (JPG 9.2 MB). Figure S7. Phylogenetic analysis of the peach RING subfamily. (JPG 8.6 MB). Figure S8. Predicted domains of HECT proteins representing each subgroup. (JPG 631.3 kb). Figure S9. Prehylogenetic analysis of the peach HECT subfamily. (JPG 920.5 kb). [file 12864_2019_6258_MOESM2_ESM.zip › Additional file 2 Figure S4.jpg]

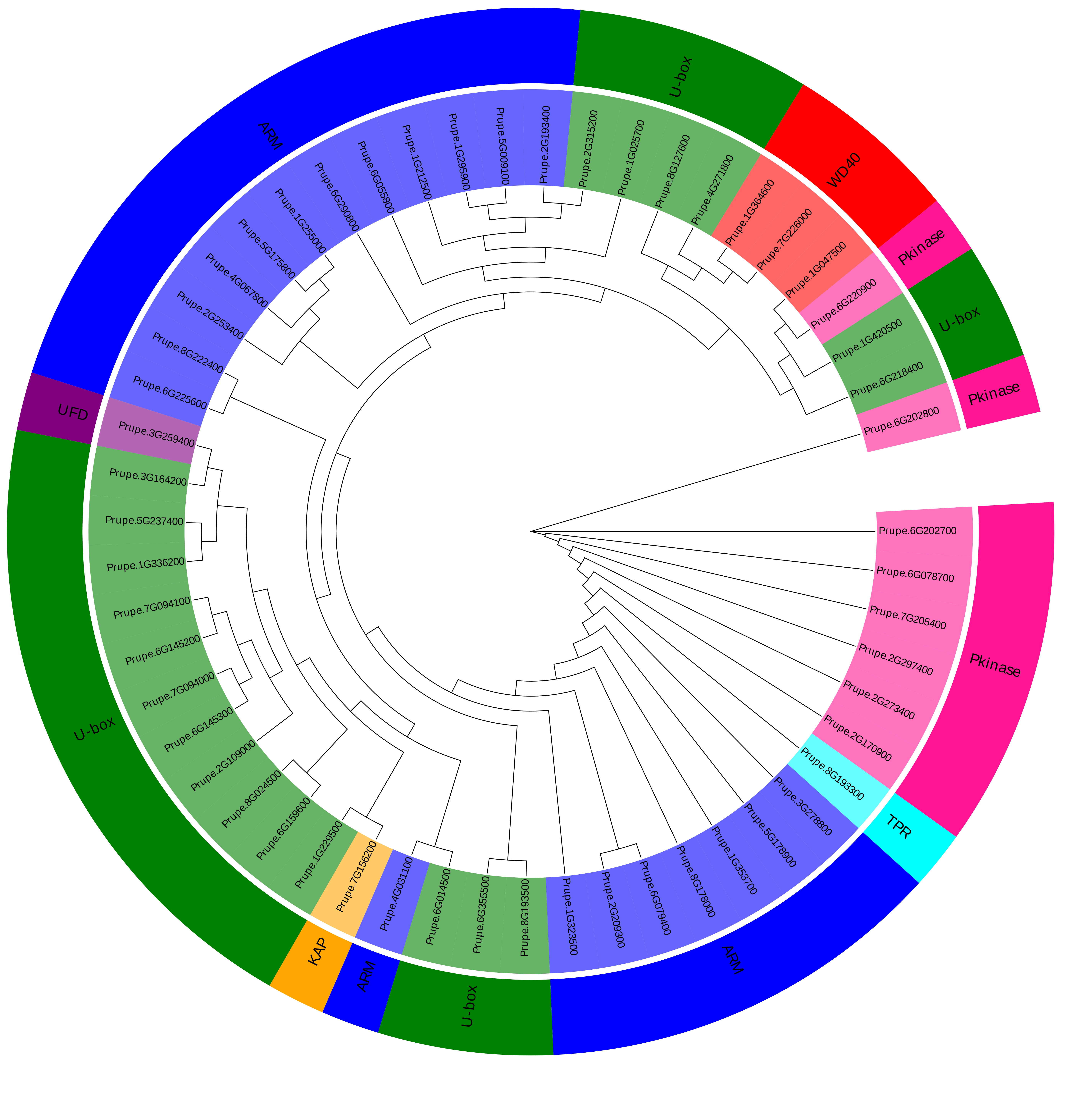

Supplement: Supplementary file 2 — Additional file 2: Figure S1. Chromosomal distribution of different PpE3 subfamilies in peach. (JPG 321 Kb). Figure S2. Predicted domains of F-box proteins representing each subgroup. (JPG 1.3 MB). Figure S3. Phylogenetic analysis of the peach F-box subfamily. (JPG 8.1 MB). Figure S4. Predicted domains of U-box proteins representing each subgroup. (JPG 2.0 MB). Figure S5. Phylogenetic analysis of the peach U-box subfamily. (JPG 3.7 MB). Figure S6. Sequence logo of the overrepresented motifs found in the RING-C2, RING-H2, RING-HC, RING-G, RING-v or RING-S/T domains of the RING proteins predicted from the peach genome. (JPG 9.2 MB). Figure S7. Phylogenetic analysis of the peach RING subfamily. (JPG 8.6 MB). Figure S8. Predicted domains of HECT proteins representing each subgroup. (JPG 631.3 kb). Figure S9. Prehylogenetic analysis of the peach HECT subfamily. (JPG 920.5 kb). [file 12864_2019_6258_MOESM2_ESM.zip › Additional file 2 Figure S5.jpeg]

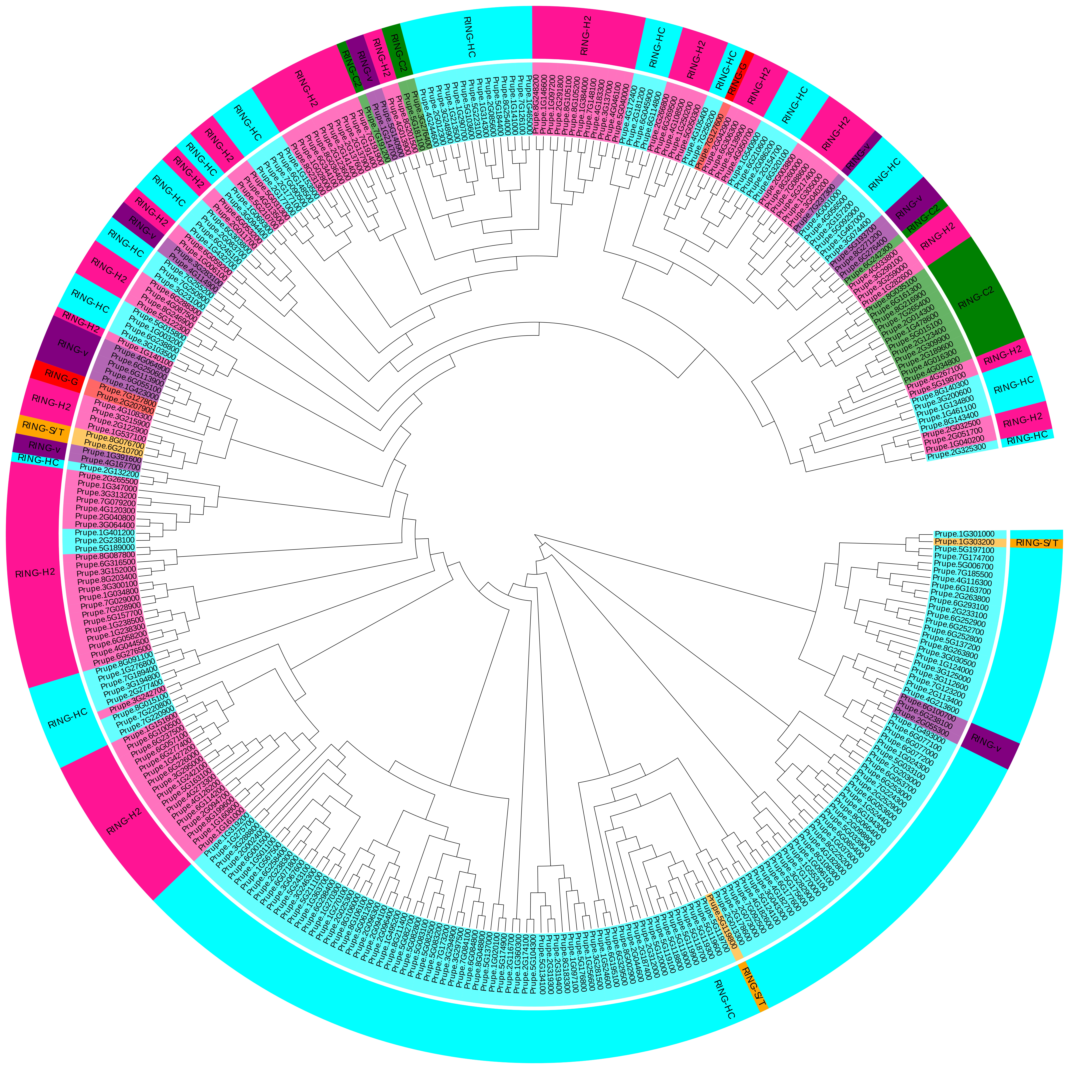

Supplement: Supplementary file 2 — Additional file 2: Figure S1. Chromosomal distribution of different PpE3 subfamilies in peach. (JPG 321 Kb). Figure S2. Predicted domains of F-box proteins representing each subgroup. (JPG 1.3 MB). Figure S3. Phylogenetic analysis of the peach F-box subfamily. (JPG 8.1 MB). Figure S4. Predicted domains of U-box proteins representing each subgroup. (JPG 2.0 MB). Figure S5. Phylogenetic analysis of the peach U-box subfamily. (JPG 3.7 MB). Figure S6. Sequence logo of the overrepresented motifs found in the RING-C2, RING-H2, RING-HC, RING-G, RING-v or RING-S/T domains of the RING proteins predicted from the peach genome. (JPG 9.2 MB). Figure S7. Phylogenetic analysis of the peach RING subfamily. (JPG 8.6 MB). Figure S8. Predicted domains of HECT proteins representing each subgroup. (JPG 631.3 kb). Figure S9. Prehylogenetic analysis of the peach HECT subfamily. (JPG 920.5 kb). [file 12864_2019_6258_MOESM2_ESM.zip › Additional file 2 Figure S7.jpg]

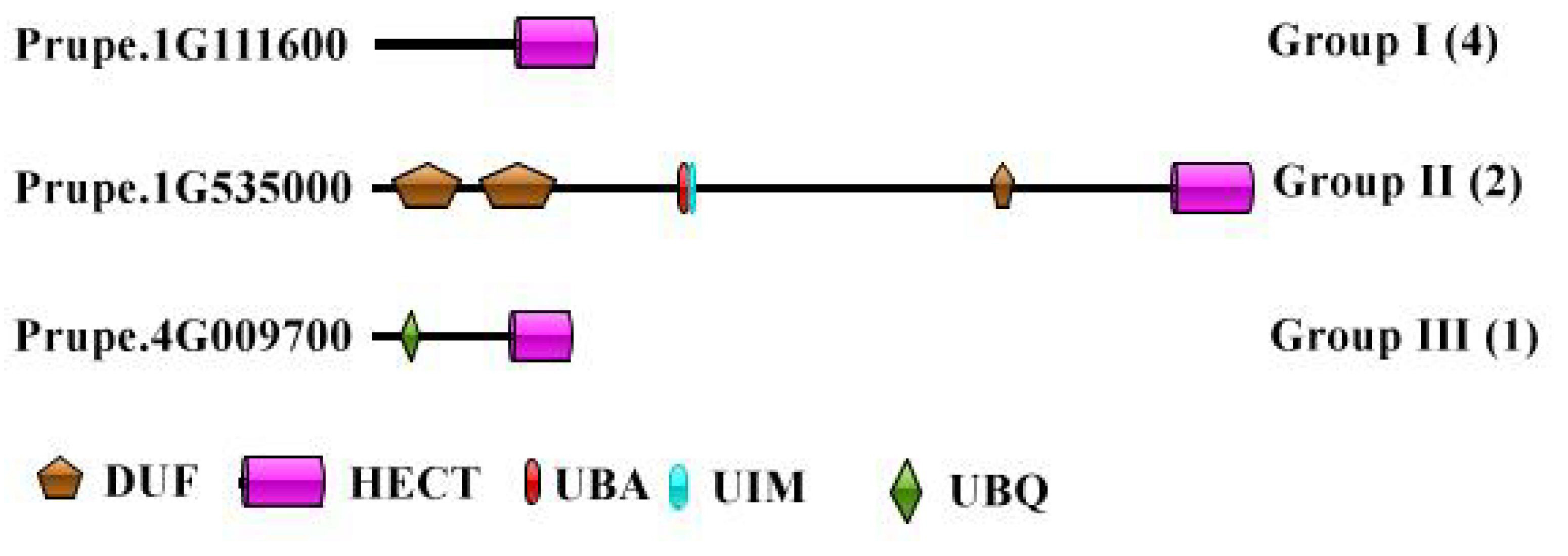

Supplement: Supplementary file 2 — Additional file 2: Figure S1. Chromosomal distribution of different PpE3 subfamilies in peach. (JPG 321 Kb). Figure S2. Predicted domains of F-box proteins representing each subgroup. (JPG 1.3 MB). Figure S3. Phylogenetic analysis of the peach F-box subfamily. (JPG 8.1 MB). Figure S4. Predicted domains of U-box proteins representing each subgroup. (JPG 2.0 MB). Figure S5. Phylogenetic analysis of the peach U-box subfamily. (JPG 3.7 MB). Figure S6. Sequence logo of the overrepresented motifs found in the RING-C2, RING-H2, RING-HC, RING-G, RING-v or RING-S/T domains of the RING proteins predicted from the peach genome. (JPG 9.2 MB). Figure S7. Phylogenetic analysis of the peach RING subfamily. (JPG 8.6 MB). Figure S8. Predicted domains of HECT proteins representing each subgroup. (JPG 631.3 kb). Figure S9. Prehylogenetic analysis of the peach HECT subfamily. (JPG 920.5 kb). [file 12864_2019_6258_MOESM2_ESM.zip › Additional file 2 Figure S8.jpg]

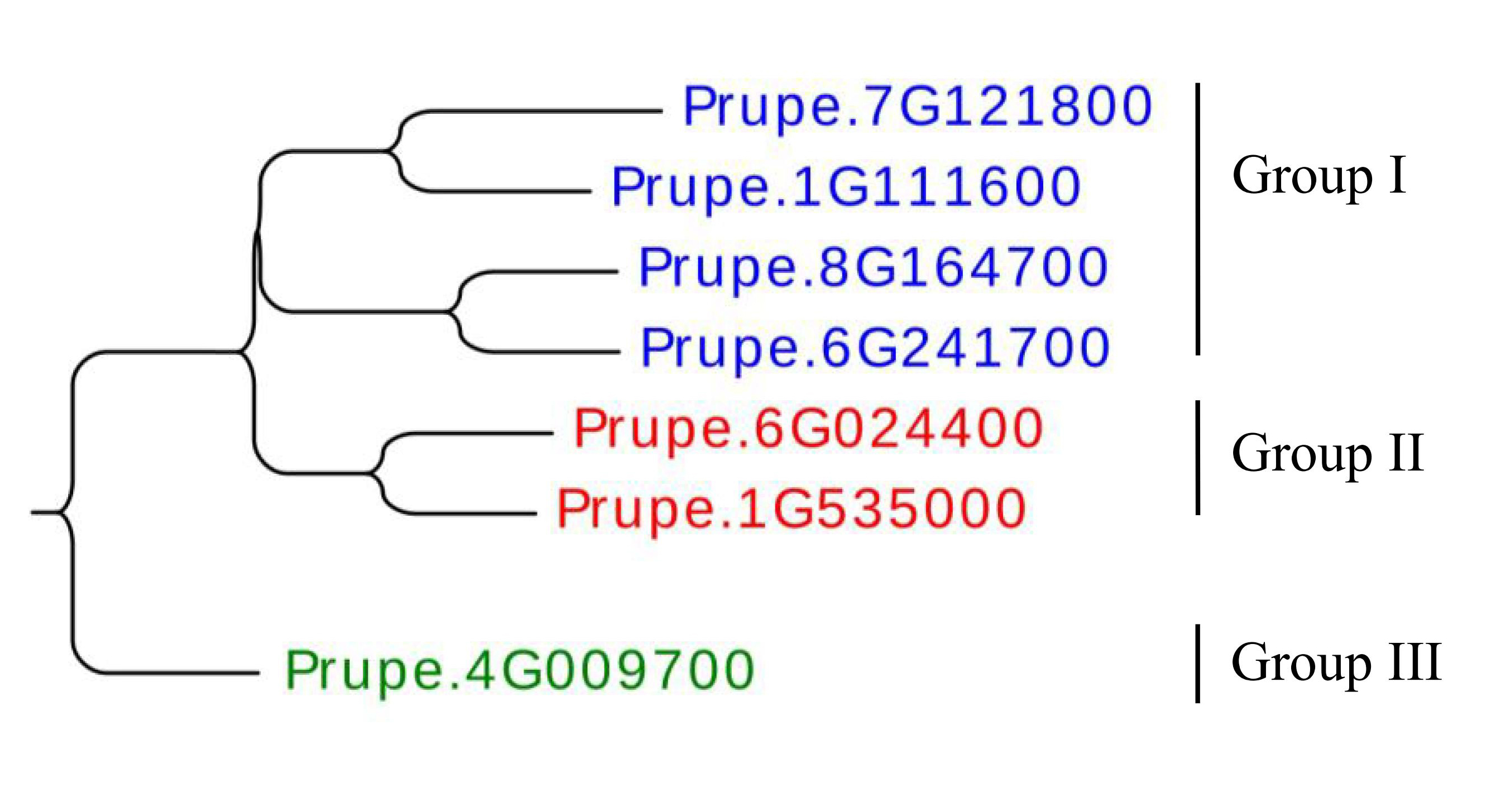

Supplement: Supplementary file 2 — Additional file 2: Figure S1. Chromosomal distribution of different PpE3 subfamilies in peach. (JPG 321 Kb). Figure S2. Predicted domains of F-box proteins representing each subgroup. (JPG 1.3 MB). Figure S3. Phylogenetic analysis of the peach F-box subfamily. (JPG 8.1 MB). Figure S4. Predicted domains of U-box proteins representing each subgroup. (JPG 2.0 MB). Figure S5. Phylogenetic analysis of the peach U-box subfamily. (JPG 3.7 MB). Figure S6. Sequence logo of the overrepresented motifs found in the RING-C2, RING-H2, RING-HC, RING-G, RING-v or RING-S/T domains of the RING proteins predicted from the peach genome. (JPG 9.2 MB). Figure S7. Phylogenetic analysis of the peach RING subfamily. (JPG 8.6 MB). Figure S8. Predicted domains of HECT proteins representing each subgroup. (JPG 631.3 kb). Figure S9. Prehylogenetic analysis of the peach HECT subfamily. (JPG 920.5 kb). [file 12864_2019_6258_MOESM2_ESM.zip › Additional file 2 Figure S9.jpg]
